# Doping in Paralympic sport: perceptions, responsibility and anti-doping education experiences from the perspective of Paralympic athletes and parasport coaches

**DOI:** 10.3389/fspor.2023.1166139

**Published:** 2023-07-07

**Authors:** Cornelia Blank, Kathrin Weber, Ian D. Boardley, Thomas Abel, Wolfgang Schobersberger, Laurie B. Patterson

**Affiliations:** ^1^ Institute for Sports Medicine, Alpine Medicine & Health Tourism UMIT TIROL—Private University for Health Sciences and Health Technology, Hall in Tirol, Austria; ^2^ School of Sport, Exercise and Rehabiliation Science, University of Birmingham, Birmingham, United Kingdom; ^3^ Institute of Movement and Neurosciences, German Sport University Cologne, Cologne, Germany; ^4^ Institute for Sports Medicine, Alpine Medicine & Health Tourism, Tirol Kliniken GmbH Innsbruck, Innsbruck, Austria; ^5^ Carnegie School of Sport, Leeds Beckett University, Leeds, United Kingdom

**Keywords:** Paralympic sport, doping, anti-doping education, Paralympic athletes, parasport coaches, responsibility

## Abstract

**Introduction:**

Limited effort has been invested in understanding doping in Paralympic sport. The limited evidence that exists suggests that factors influencing doping in parasport are similar to Olympic sport. However, based on the design and nature of the previous studies, where methods have been mostly limited to qualitative data and prevalence numbers, further research is warranted to extend previous findings.

**Methods:**

Informed by current evidence from Paralympic and Olympic sport, we aimed to investigate (1) para-athletes' perceptions of Anti-Doping Rule Violations (ADRVs) and responsibility for them, (2) descriptive norms for doping in parasport (3) perceptions of anti-doping education and legitimacy of anti-doping authorities, and (4) coach engagement in doping prevention and levels of doping confrontation efficacy using a quantitative survey approach.

**Results:**

In total, valid survey responses from 126 Paralympic athletes and 35 coaches from four countries (Germany, Austria, Switzerland, UK) were analysed for experience with anti-doping, descriptive norms, anti-doping education, perceived legitimacy, knowledge, and doping confrontation efficacy (coaches only). Across both athletes and coaches, the level of education was generally good and doping willingness was low. Classification cheating was considered a form of doping and seems to be an important issue for athletes and coaches, especially within the UK sample. For 33.3% of the athletes, doping control was their first experience with anti-doping. Coaches' engagement with doping prevention activities and their perceived efficacy to confront doping-related matters appears to be higher compared to Olympic coaches' samples.

**Discussion:**

Sport organisations/NADOs in Paralympic sport could use synergies with those organisations in Olympic sport, adopting similar approaches to anti-doping education, also focusing on a balanced communication of doping prevalence numbers and testing figures. Efforts to ensure athletes are educated about anti-doping before they are tested should be upheld. It seems that in para sport, different compared to able-bodied coaches, anti-doping organizations do not have to convince the coaches about their roles (i.e., being responsible for anti-doping education) anymore but can directly build on these resources. Overall, it seems that there are few differences between parasport and able-bodied sports and thus responsible organisations could use the existing programmes in Olympic sport and only adapt special content (e.g., boosting) which is unique to Paralympic athletes.

## Highlights

• Doping is perceived as highly prevalent in Paralympic sport.
• 33% of para-athletes experienced anti-doping control as their first contact with the anti-doping system compared to 60% for education.
• Parasport coaches take their roles and responsibilities seriously and have good anti-doping knowledge.
• Cheating on classification was perceived as being akin to doping and as a significant integrity issue in Paralympic sport.

## 1. Introduction

Doping is recognized as a serious problem in organised sport (1–3). Research traditionally focused on doping in able-bodied sport contexts. Yet, disabled elite sport also represents a part of the organised sport community. Even though it is a smaller sport community compared to Olympic sport, doping occurs and is an existing issue (4–7). Annual reports of the World Anti-Doping Agency (WADA) between 2013 and 2019 outline 252 Anti-Doping Rule Violations (ADRVs) in sport disciplines which are overseen by the International Paralympic Committee (IPC) (8–14). In detail, ADRVs between 2013 and 2019 were 22, 21, 33, 26, 18, 39, and 93 respectively. The testing figures were 2.284, 3.317, 3.689, 5.175, 4.220, 5.867, and 6.805 respectively leading to a % proportion of ADRVs of 0.9%, 0.6%, 0.9%, 0.5%, 0.4%, 0.6%, and 1.4% respectively. Even though the absolute numbers of ADRVs are low, they indicate an increase compared to the 60 ADRVs in total that occurred between 2000 and 2011 (5). Additionally, as in Olympic sport, the true prevalence is likely to be higher due to an expected underestimation of doping by pure testing figures (15–17). Doping can have severe consequences for the athletes as well as their entourage (e.g., coaches). An ADRV can not only result in sanctions such as bans from sport (18) and legal consequences (19) but evidence also indicates negative physiological [e.g., osteoporosis (20)], psychological [e.g., depression (21)] and financial [e.g., fines (22)] effects. Consequently, doping prevention should be considered an important activity for all stakeholders in Olympic and Paralympic sport to maintain athletes' health and well-being as well as clean, fair sport (18).

Prevention, as defined in a public health context (23), aims to decrease the likelihood of a specific undesired outcome through specific, targeted measures. Those measures should be based on evidence-based information about risk factors increasing the likelihood of the outcome, but also, about protective factors, that decrease the likelihood of the outcome. The latter perspective is rooted in the concept of health promotion (23). Research in Olympic sport has addressed both with respect to doping (24–26) and researchers acknowledged that doping behaviour is related to a complex, multi-layered network of risk but also protective factors (2, 27). Although connected, these factors represent different areas, including personal variables [e.g., attitudes, norms, values (24, 25)], interpersonal factors [e.g., athlete support personnel (ASP) (28–30)], and contextual/environmental factors [e.g., training group (28), injury (31)]. After 20 years of research, it appears that in Olympic sport, some of the most important factors include the use of nutritional supplements (32), perceived social norms, and positive attitudes toward doping (24, 25). Additionally, it was shown that the athlete's direct environment, especially coaches, plays an important role in an athlete's decision to dope (or not) (26, 33, 34).

To support athletes and those around them, including coaches, in creating and sustaining drug-free environments, WADA introduced the World Anti-Doping Code (WADC) and its associated International Standards. The International Standard of Education (35) requires education, including values-based programmes, from its signatories to address risk and protective factors. The ISE specifies that a range of target groups, such as coaches, should be included in educative measures. However, up to date, most signatories include information-based education and do not tackle variables such as norms and values (36). Recently, educational material to address morality and moral disengagement, as part of values-based education was developed and tested (37). However, evaluations of education programmes are rare. Moreover, even though not specifically stated, most existing education programs (whether evaluated or not) mainly refer to able-bodied sport and are based on evidence gathered in able-bodied sport samples. Therefore, it is difficult for National Anti-Doping Organisations (NADOs) and international federations (IFs) to design and deliver evidence-informed, tailored, and targeted education programmes, as required by the ISE.

Unfortunately, limited effort has been invested in understanding doping in Paralympic sport and athletes’ and coaches' perceptions of it to date. One exception to this is a recent qualitative study that generated an in-depth picture of athletes' and coaches' perceptions of doping in Paralympic sport as well as relevant risk and protective factors (6, 7). This study suggests that factors influencing doping in parasport might be similar to Olympic sport, such as a lack of knowledge and education (5–7, 38), increasing pressure due to professionalism and financial incentives (6, 7, 39), the risk of injuries (39, 40), and the existence of un-harmonized rule implementation and resulting “loopholes” in the system (6, 7). However, based on the limited number and specific design (e.g., qualitative methods used with small samples in few countries) of the previous studies, further research is warranted to extend these findings.

Thus, the aim of this study was to test the validity of some of these earlier findings with a larger sample of Paralympic athletes and coaches through a quantitative approach. Informed by current evidence from Paralympic and Olympic sport, we aimed to investigate (1) para-athletes' perceptions of what constitutes an ADRV (i.e., to assess the importance of classification cheating) and who is responsible for them, (2) descriptive norms for doping in parasport (i.e., to assess whether it is considered even relevant) (3) perceptions of anti-doping education and legitimacy of anti-doping authorities (i.e., to assess if para-athletes have different educational needs and different legitimacy perceptions due to different needs), and (4) coach engagement in doping prevention and levels of doping confrontation efficacy (i.e., coaches' ability to effectively confront athletes whom they suspect of doping). Taking an explorative and descriptive approach, this is the first survey-study with para-athletes that we are aware of that goes beyond the level of knowledge and prevalence numbers with respect to doping. We aimed to provide a clearer picture of perceptions of doping in parasport that would inform the work of policymakers and education providers. To ensure that programmes are as tailored and targeted as possible (in line with the ISE), we also aim to undertake analyses comparing different socio-demographic variables (e.g., sex, age, sport). From a scientific perspective, we intended to broaden the evidence base to define theory-driven hypotheses to test in future studies.

## 2. Materials and methods

### 2.1. Study design and research instrument

To address our research aims, an online survey was designed to investigate the following overarching themes: (1) characteristics of the sample, (2) descriptive norms, (3) anti-doping education, legitimacy and perceived knowledge of doping-related issues, and (4) coaches' engagement in doping prevention, roles and responsibilities within the WADC, and doping confrontation efficacy. If available, validated scales (details below) were used and checked for reliability in our sample using Cronbach's Alpha. Additional questions were designed and added when we felt available scales could not capture the required information. The anonymous survey was available in German, English, and French and took 20–30 min to complete. Where necessary, parts of the survey were translated by a professional translating office or native-speaking researchers (including independent back-translation) (41) to avoid language bias.

#### 2.1.1. Sample characteristics and experience with anti-doping

To describe the sample, socio-demographic variables, as well as testing history, perception of ADRVs, responsibility of ADRVs, and doping willingness, were assessed. Socio-demographic items referred to sex, age, country, disability, sport discipline, highest level of sporting competition/highest level of their athletes' sport competition for coaches, years of experience/coaching on their highest level, and if they consider themselves a professional athlete/coach. “Professional” was defined as the athlete/coach being able to make a living due to sport funding, sponsorship deals and/or payments/salaries related to their sport career; respondents were classified as professional or not professional based on their response. Associated to that, we asked athletes only (1 = strongly disagree; 7 = strongly agree) if being an elite athlete increases monetary incentives (e.g., make a living) to do the sports.

To assess experience with anti-doping, we asked athletes if they had participated in a doping control before. With respect to the perception of an ADRV, we asked coaches and athletes to rate on a 7-point scale (1 = strongly disagree; 7 = strongly agree) if given statements (e.g., taking nutritional supplements) represent a form of doping. Athletes and coaches had the chance to add other statements and rate them. In terms of ADRV responsibility, we asked to what percentage they thought the presented stakeholders (e.g., athlete, coach, family members, other) can be held responsible for a positive result from a doping control test. We assessed doping willingness based on Stanger, Whitaker (42), providing athletes with eight scenarios and asked about their willingness to use a banned substance or method, e.g., if it increased your chances to gain a professional contract or funding (1 = not at all willing; 5 = extremely willing; Cronbach's-α = .95).

#### 2.1.2. Descriptive norms

Adapted from Barkoukis, Lazuras (43), we asked athletes and coaches what percentage (out of 100%) of elite athletes engage in doping. Eight different statements were presented with focus on Paralympic sport in general/in their country/in their own sport discipline, concerning different sport disciplines (strength sport, endurance sport, skilled sport) and able-bodied elite sport in general/in their country.

#### 2.1.3. Anti-doping education, legitimacy, and perceived knowledge of doping-related issues

Athletes and coaches were asked (1) what their first experience with the anti-doping system was, (2) how many educational sessions they attended in the last year (none—more than 10), (3) if they perceive the received anti-doping education as trustworthy (coaches were also asked if they perceived it as “worthwhile”) (yes/no), (4) what could be included in case they think the received education was not trustworthy/worthwhile (open question), and (5) where they got their anti-doping information from (multiple answers possible, e.g., NADO, WADA, IPC). In addition, coaches were asked if they received anti-doping education in their coaching career (primary education, secondary education, none, other), and could state what they found helpful within the anti-doping education (open question). In more general terms, we were also interested in the perceived legitimacy of sports authorities entrusted with anti-doping. Informed by Woolway, Lazuras (44), athletes and coaches were asked to indicate the level of agreement (1 = strongly disagree; 7 = strongly agree; Cronbach's-α_athletes_ = .90; Cronbach's-α_coaches_ = .83) with nine statements, e.g., the current anti-doping system is effective in protecting clean sport.

Based on the WADC 2021 (18) and WADA's International Standard for Education (ISE) (35), athletes were asked to rate if they felt well informed (1 = strongly disagree; 7 = strongly agree) about 12 topics, e.g., the consequences of doping, including sanctions, health, and social consequences (Cronbach's-α = .93). Coaches were asked with one item how well they felt informed about their rights and responsibilities according to the current WADC (1 = not at all informed; 7 = very well informed).

#### 2.1.4. Coaches: engagement in doping prevention, roles, and responsibilities within the WADC, and doping confrontation efficacy

Informed by previous research in Paralympic and Olympic sport, we added three areas of interest specific to coaches. With respect to coach engagement in doping prevention, we asked six questions such as “do you prepare your athletes for doping controls?” and “is doping and doping prevention a relevant topic in your training routine?” to be answered in a yes/no format. We were further interested in their agreement with their roles and responsibilities as outlined in the WADC [2021 (18);] and asked them six statements to be rated (1 = strongly disagree; 7 = strongly agree) with each, e.g., I use my influence on athlete's values and behaviour to foster anti-doping attitudes. To evaluate coaches' confidence in their abilities to confront athletes on doping matters, we used the Doping Confrontation Efficacy Scale (DCE) (45). This scale includes five subscales (1 = initiation; Cronbach's-α = .81, 2 = intimacy; Cronbach's-α = .76, 3 = legitimacy, Cronbach's-α = .90; 4 = outcomes, Cronbach's-α = .77; 5 = resources, Cronbach's-α = .82) with four questions each on a 7-point scale (1 = no confidence; 7 = complete confidence). Initiation represents coaches' beliefs in their ability to confront athletes regarding doping issues and establish the purpose for the confrontation. Legitimacy reflects their belief in their ability to establish valid grounds for establishing a confrontation. Personal resources pertains to the degree to which coaches believe they have the requisite resources (i.e., time, energy, and information) to cope effectively with the cognitive and emotional demands of confrontations. Intimacy relates to coaches' perceived ability to confront athletes without appearing judgmental. Finally, expected outcomes reflect coaches' beliefs in their ability to confront athletes regardless of possible resulting positive and negative outcomes.

Coaches and athletes could provide further feedback on the survey in an open-end format on the final page. This feedback is integrated into the results section where applicable. To receive the full survey, please contact the corresponding author.

### 2.2. Procedure and participants

The current study is part of a bigger research project aiming to explore both Paralympic athletes' and parasport coaches’ doping-related perceptions, reasons, and knowledge. Both athletes and coaches received a written online information document about the research itself as well as data protection and contact details. After reading that information, they had to confirm consent before they were able to start the survey. The study was approved by the first author's university's ethics board (RCSEQ 2801/20).

Participants included professional Paralympic athletes and coaches fitting the following criteria. Athletes who: (a) were 18 years or older, (b) were pursuing a sport recognized by the IPC, (c) met the IPC's definition of a Paralympic athlete (46), (d) had participated for five or more years in national or international competitions, and (e) were registered within an anti-doping testing pool. Coaches who (a) were 18 years or older, (b) coached disabled elite athletes in a sport recognized by the IPC, (c) had coached five or more disabled elite athletes for national or international competitions for five years or more, and (d) were officially registered as an eligible coach by their respective sport federation.

To address as many Paralympic athletes and coaches as possible, we followed two main approaches. First, we contacted parasport organisations/clubs (*n *= 169) in the United Kingdom, Germany, Austria, and Switzerland and asked them to help us distribute the survey among their athletes and coaches. We received great support from the National Paralympic Committee of Germany, the Austrian Paralympic Committee, and PluSport Disabled Sports Switzerland. Second, we listed all athletes/coaches being presented in the national squads on the sport organisations' webpages and invited them personally via public email addresses and/or social media accounts (i.e., Instagram and/or Twitter). In total, 884 Paralympic athletes and 127 parasport coaches were identified, of which we were able to contact 644 athletes and 110 coaches between March 2021 and February 2022. Data collection was prolonged due to the delayed Summer Paralympics in 2021, as we paused data collection for the Games.

### 2.3. Data analysis

All data were analysed with the software programme SPSS 27.0 (Statistical Package for the Social Sciences, Chicago, Illinois). As we used slightly different surveys for athletes and coaches, we analysed them separately. All variables were descriptively analysed using frequencies and/or mean ± standard deviation (SD). As outlined above, scales were built if appropriate (Cronbach's Alpha > 0.7), and a mean sum score was computed and presented with its respective SD. To analyse differences between age and sex regarding athletes'/coaches' country, we used ANOVA/Welch (depending on the homogeneity of variance) (age) and chi-square tests (sex). Two-sided significance level was set at *p* < 0.05.

## 3. Results

### 3.1. Sample characteristics

#### 3.1.1. Socio-demographic information

After cleansing the data set, we analysed 126 (out of 175) valid data sets for athletes. Of the 126 athletes, 36.5% were female with no significant difference in the proportion of females represented between the countries (*p* = 0.69). Athletes' age was significantly different between the four countries (*p* = 0.01; eta^2 ^= 0.12) with the oldest being Austrian (36.86 years ± 13.2) and the youngest being German (28.8 ± 8.2). Five outliers (≥ 56 years) were identified from Para Archery (*n* = 1), Wheelchair Tennis (*n* = 1), Wheelchair Fencing (*n* = 1), and Para Cycling (*n* = 2). In terms of sport, the sample represented 21 of 28 IPC acknowledged sport disciplines. In terms of disability, we could not classify thirteen athletes into one of the IPC classifications as for seven, the information about their disability was too general/vague (e.g., neurological condition) and six athletes gave no answer (for the classification of the rest, refer to Table 1). In total, 60 athletes (47.6%) defined themselves as “professional” (for more details, refer to Table 1).

**Table 1:** Sociodemographic data of Paralympic athletes and parasport coaches.

Sex (%)	Athletes		Coaches	
	Female (*n* = 46)	Male (*n* = 80)	Female (*n* = 5)	Male (*n* = 30)
UK	30.4	27.5	20.0	10.0
Germany	32.6	25.0	80.0	50.0
Austria	19.6	23.8	-	10.0
Switzerland	17.4	23.8	-	30.0
Sport disciplines (%)^a^	Athletes (*n* = 123)		Coaches (*n* = 35)	
Cycling	18.7		-	
Wheelchair basketball	13.8		8.6	
Athletics	9.8		17.1	
Swimming	-		22.9	
Triathlon	-		8.6	
Competition level (%)	Athletes (*n* = 126)		Coaches (*n* = 35)	
International	94.4		94.3	
Paralympic Games	63.5		94.3	
Disability (%)	Athletes (*n* = 125)			
Since birth	49.2		-	
Through incident	50.0		-	
Classification of disability^1^ (%)	Athletes (*n* = 126)			
Impaired muscle power	42.1		-	
Limb deficiency	16.7		-	
Vision impairment	7.1		-	
Age (M ± SD)	Athletes (*n* = 126)		Coaches (*n* = 35)	
Total sample	32.0 ± 10.6		48.7 ± 13.4	
UK	28.9 ± 5.7		46.0 ± 12.3	
Germany	28.8 ± 8.2		45.1 ± 12.2	
Austria	36.9 ± 8.2		50.3 ± 12.7	
Switzerland	35.1 ± 12.9		57.2 ± 14.6	
Years competing/coaching on highest level (M ± SD)	9.7 ± 5.6		11.2 ± 6.0	

^a^ Top three.

In total, 35 (out of 49) valid datasets for coaches were analysed and 14.0% of the sample were female (*n* = 5) with no significant difference between the countries (*p* = 0.38). Nearly 25% of coaches (*n* = 9) were ≥ 60 years with no significant differences between the countries (*p* = 0.15; eta^2 ^= 0.15); female coaches (*M* = 47.6 ± 14.2) were similar in age to male (M = 48.9 ± 13.5 years) coaches. They represented 16 out of 28 IPC sport disciplines and 68.6% were classified as professional (for more details, refer to Table 1). Due to the skewed distribution of coaches’ sex and due to the low number of female coaches, no sex-related sub-analyses were performed for the outcome variables.

#### 3.1.2. Perception of ADRV, testing history, and responsibility of ADRV

With respect to what is perceived to represent a form of doping (refer to Table 2), both athletes and coaches, except for UK coaches, indicated the highest agreement with the statement of misuse of prohibited substances and/or methods. UK coaches indicated the highest agreement with classification cheating, which had the second highest agreement for the rest of the respondents. In the open-ended section of the questionnaire, several athletes additionally commented on “*classification doping”* that it “*is a far bigger issue (…) than drug doping”* and it should “*gain even more importance”*. One athlete criticized the IPC and international federations for their handling of this issue. UK athletes and coaches, in general, had the highest agreement scores in all items except “taking nutritional supplements”. Austrian athletes and Swiss coaches, in general, had the lowest agreement indicators with a mean agreement <4 (Austrian athletes) and <3 (Swiss coaches). We found some significant differences between the countries. UK athletes had a significantly higher agreement to substance use (*p* = 0.01), classification cheating (*p* = 0.02), and misuse of medication (*p* = 0.006) compared to Austrian athletes. UK coaches had a significantly higher agreement to classification cheating compared to German (*p* = 0.004) and Swiss Coaches (*p* = 0.007).

**Table 2:** Athletes’ and coaches’ perception of what represents a form of doping^a^ and whom they consider to be responsible.

	Total sample		UK		Germany		Austria		Switzerland	
	(M ± SD)		(M ± SD)		(M ± SD)		(M ± SD)		(M ± SD)	
Perception of ADRV (Likert Scale from 1 = do not agree at all—7 = completely agree)										
	Athletes	Coaches	Athletes	Coaches	Athletes	Coaches	Athletes	Coaches	Athletes	Coaches
Using banned substances or methods as listed on the WADA Prohibited List	5.1 ± 2.7	4.2 ± 2.9	5.9 ± 2.3^b^	6.0 ± 2.0	5.2 ± 2.7	4.5 ± 3.0	3.7 ± 2.9	5.0 ± 3.5	5.3 ± 2.6	2.6 ± 2.4
Cheating on classification^b^	4.7 ± 2.7	4.2 ± 2.9	5.6 ± 2.4^d^	7.0 ± 0.0^c,e^	4.7 ± 2.7	4.2 ± 3.0	3.5 ± 2.8	4.7 ± 3.2	4.8 ± 2.6	2.7 ± 2.6
Misusing medications	4.6 ± 2.7	3.8 ± 2.8	5.4 ± 2.3^d^	5.5 ± 1.7	4.7 ± 2.7	3.8 ± 3.0	3.1 ± 2.6	5.0 ± 3.5	4.8 ± 2.6	2.4 ± 2.3
Boosting	4.1 ± 2.4	3.3 ± 2.5	4.4 ± 2.4	5.0 ± 2.2	4.1 ± 2.2	3.3 ± 2.6	3.3 ± 2.6	4.0 ± 2.6	4.6 ± 2.5	2.4 ± 2.3
Consuming marijuana prior to competition	3.7 ± 2.5	3.7 ± 2.8	4.4 ± 2.4	4.5 ± 3.0	3.5 ± 2.4	3.9 ± 3.0	2.9 ± 2.6	4.0 ± 3.0	4.0 ± 2.5	2.7 ± 2.6
Taking nutritional supplements^b^	2.7 ± 1.7	3.3 ± 1.7	2.2 ± 1.8	2.8 ± 1.7	2.9 ± 1.5	3.5 ± 2.0	3.2 ± 1.6	3.3 ± 1.2	2.8 ± 1.9	3.1 ± 1.5
Consuming alcohol prior to a competition	2.8 ± 2.2	2.4 ± 2.1	2.8 ± 2.1	2.8 ± 2.2	3.0 ± 2.1	2.7 ± 2.2	2.5 ± 2.3	3.7 ± 3.1	2.8 ± 2.2	1.4 ± 1.0
Consuming caffeinated drinks prior to a competition^b^	2.7 ± 1.8	2.6 ± 1.4	2.2 ± 1.8	1.8 ± 1.0	2.5 ± 1.7	2.6 ± 1.5	3.4 ± 2.0	2.7 ± 0.6	2.7 ± 1.7	2.8 ± 1.6
Perception about responsibility for ADRV (out of 100%)^a^										
	Total sample (M ± SD)		UK (M ± SD)		Germany (M ± SD)		Austria (M ± SD)		Switzerland (M ± SD)	
	Athletes	Coaches	Athletes	Coaches	Athletes	Coaches	Athletes	Coaches	Athletes	Coaches
Athlete	75.5 ± 30.9	74.3 ± 28.8	90.3 ± 22.7^c,d^	60.3 ± 45.0	71.0 ± 30.4	75.4 ± 26.9	62.2 ± 36.4	93.3 ± 11.5	75.0 ± 28.0	71.8 ± 29.3
Coach	37.6 ± 30.6	41.8 ± 26.6	43.2 ± 37.1	33.8 ± 26.3	35.1 ± 28.0	39.3 ± 18.4	41.1 ± 30.8	59.7 ± 39.5	29.4 ± 22.7	45.3 ± 40.0
Other ASP	30.5 ± 30.5	33.1 ± 26.8	42.2 ± 38.9^e^	39.0 ± 36.6	25.3 ± 24.9	33.5 ± 23.3	32.1 ± 28.9	16.3 ± 19.6	19.9 ± 20.5	35.5 ± 33.5
Sport federation	18.8 ± 26.0	20.7 ± 27.1	33.6 ± 37.1^c,e^	45.5 ± 36.2	11.8 ± 15.8	22.4 ± 27.9^f^	16.4 ± 17.1	0.7 ± 0.6	8.7 ± 12.7	11.0 ± 15.0
Family members	13.4 ± 23.2	11.8 ± 17.6	26.4 ± 33.7^c,e^	13.0 ± 24.7	6.9 ± 15.6	14.6 ± 19.4	10.6 ± 15.2	9.7 ± 14.2	7.0 ± 12.7	5.5 ± 10.4
Sport club	14.3 ± 23.2	9.3 ± 12.4	27.6 ± 36.4^e^	6.0 ± 4.9	8.6 ± 13.0	14.4 ± 15.8^f^	11.3 ± 14.4	0.7 ± 0.6	8.0 ± 10.4	5.4 ± 5.8

^a^ As the number of athletes and coaches differed within the questions, *n* is not stated.

^b^ No Anti-Doping Rule Violation according to the IPC Anti-Doping Code.

^c^ Significant difference compared to Germany.

^d^ Significant difference compared to Austria.

^e^ Significant difference compared to Switzerland.

^f^ Significant difference compared to Austria.

Overall, athletes were the first to be held responsible for an ADRV as indicated by all respondents, when ranked in comparison with other potential stakeholders, with UK athletes agreeing most strongly with that statement (90.3%). This agreement was significantly higher compared to Germany (*p* = 0.03) and Austria (*p* = 0.007). In general, UK athletes had a slightly different answer pattern (i.e., higher agreement) compared to Germany and Switzerland in terms of sport federations and families' responsibility (for details, refer to Table 2). Except for UK coaches, all other athletes and coaches rated the coach as second to be held responsible—for UK coaches, the coach had the fourth highest rating, after the sport federation and other ASP. Other responsible stakeholders mentioned by athletes and coaches were teammates, sponsors, and wider social pressure. For details, see Table 2.

#### 3.1.3. Doping willingness

Overall, athletes' score of doping willingness was *M* = 1.16 ± 0.51 with no significant differences between the countries (*p *= 0.56, eta^2 ^= 0.02). No significant differences were found between male (*n *= 78) and female (*n *= 44) athletes (1.14 ± 0.4 vs. 1.21 ± 0.7; *p *= 0.58, *d *= 0.1), whether they were professional (*n *= 58) or not (*n *= 64; 1.17 ± 0.6 vs. 1.16 ± 0.4; *p *= 0.91, *d *= 0.02), competed at Paralympic Games (*n *= 78) or not (*n *= 37; 1.16 ± 0.5 vs. 1.14 ± 0.4; *p *= 0.86, *d *= 0.04) or are impaired since birth (*n* = 59) or through an incident (*n *= 62; 1.16 ± 0.6 vs. 1.18 ± 0.5; *p *= 0.85, *d *= 0.04). Difference tests by sport discipline were not feasible due to the low number of athletes in each discipline. Also, no age-related correlation was found (*p *= 0.69, *r *= −0.04).

### 3.2. Descriptive norms

Athletes and coaches rated the doping prevalence higher in Olympic sports compared to Paralympic sports. In Paralympic sport, strength and endurance sports were ranked higher than team skill sport, and the expected doping prevalence in one's own Paralympic sport was rated the second lowest. No specific patterns were identified with respect to the countries and no significant differences were found. Overall, out of the eight items, six showed a different pattern for male (*n *= 80) and female (*n *= 46) athletes, where females estimated the prevalence significantly higher compared to males. Those athletes who were impaired since birth estimated the doping prevalence higher compared to those who were impaired due to an incident for two out of the eight items. No differences were found related to being professional or not (0.15 < *p *< 0.83; 0.04 <*d < *0.26), or participating in Paralympic Games or not (0.12 < *p *< 0.92; 0.01 <*d < *0.32). No age-related correlations were found. For details, refer to Table 3.

**Table 3:** Athletes’ and coaches’ perception of doping prevalence in various populations (%)^a.^

	Total sample		UK		Germany		Austria		Switzerland	
	(M ± SD)		(M ± SD)		(M ± SD)		(M ± SD)		(M ± SD)	
	Athletes	Coaches	Athletes	Coaches	Athletes	Coaches	Athletes	Coaches	Athletes	Coaches
International level Olympic sport^b^	30.6 ± 22.4	31.2 ± 21.2	32.5 ± 22.6	23.8 ± 19.0	30.6 ± 21.1	37.4 ± 20.9	34.4 ± 25.2	17.7 ± 17.8	24.5 ± 20.9	26.1 ± 22.8
International level Paralympic strength sports (e.g., weightlifting)	28.9 ± 23.6	34.8 ± 26.8	28.8 ± 25.9	28.0 ± 38.3	27.5 ± 22.9	39.9 ± 24.1	31.7 ± 22.6	30.3 ± 31.0	28.2 ± 23.5	29.6 ± 28.3
International level Paralympic endurance sports (e.g., cycling)^c,h^	21.9 ± 20.2	27.0 ± 22.8	22.8 ± 23.7	17.3 ± 22.0	21.4 ± 20.4	33.1 ± 22.3	25.9 ± 20.1	20.0 ± 17.3	17.1 ± 14.5	21.6 ± 26.6
International level Paralympic sport^d^	19.8 ± 18.0	23.3 ± 15.5	22.3 ± 22.4	19.5 ± 20.3	17.1 ± 13.6	26.2 ± 13.1	22.6 ± 20.6	18.0 ± 19.2	16.9 ± 12.8	21.4 ± 19.4
International level Olympic sport in your country^e^	19.4 ± 19.5	15.1 ± 12.5	20.2 ± 23.2	14.5 ± 23.7	17.5 ± 15.0	15.9 ± 10.2	23.6 ± 21.5	12.3 ± 12.7	16.4 ± 17.0	14.8 ± 12.6
International level Paralympic sport in your country^f^	14.9 ± 19.6	7.8 ± 10.9	10.2 ± 20.7	12.8 ± 24.8	8.6 ± 8.3	6.7 ± 4.6	10.8 ± 19.0	8.7 ± 11.0	5.0 ± 10.8	7.6 ± 13.4
At an international level in your Paralympic sport^g^	8.8 ± 15.7	16.4 ± 15.9	15.1 ± 24.7	16.3 ± 22.7	12.5 ± 15.2	19.1 ± 16.6	16.6 ± 20.8	5.7 ± 3.1	15.5 ± 15.4	14.3 ± 13.7
International levelParalympic coordination skilled sport (e.g., judo)^i^	10.1 ± 15.5	12.8 ± 13.4	12.4 ± 21.8	12.3 ± 16.3	10.0 ± 11.9	15.6 ± 14.1	11.3 ± 15.4	4.3 ± 1.2	5.6 ± 7.3	10.1 ± 13.6

^a^ As the number of athletes and coaches differed within the questions, we did not state *n*.

^b^ Significant differences between male and female athletes. In detail: 39.64 ± 23.93 vs. 26.68 ± 20.02; *p* = 0.001.

^c^ 27.95 ± 22.34 vs. 26.33 ± 22.34; *p* = 0.03.

^d^ 24.30 ± 22.16 vs. 17.30 ± 14.87; *p* = 0.04.

^e^ 28.24 ± 24.35 vs. 14.64 ± 14.21; *p* = 0.002.

^f^ 12.67 ± 20.92 vs. 6.69 ± 11.53; *p* = 0.046.

^g^ 20.88 ± 26.12 vs. 11.65 ± 14.14; *p* = 0.04.

^h^ Significant differences between athletes who are impaired since birth compared to those who are impaired due to an accident. In detail: 26.01 ± 23.68 vs. 17.93 ± 15.37; *p* = 0.03.

^i^ 13.36 ± 19.76 vs. 7.02 ± 9.14; *p* = 0.03.

### 3.3. Anti-doping education, perceived knowledge, and legitimacy of sport authorities entrusted with anti-doping issues

For many athletes (59.5%), education was their first contact with the anti-doping system, whereas 33.3% experienced a doping control first. Likewise, 68.6% of coaches attended a form of anti-doping education as their first contact with anti-doping, whereas 20.0% experienced a doping control of one of their athletes as their first contact. Around two-thirds, 60.3% of the athletes and 62.9% of the coaches, received 1–3 education sessions within the last year; 28.6% of athletes and 22.9% of coaches had not received any kind of anti-doping education. For the coaches, most of them (60%) indicated to have received the education as part of their secondary education and training, whereas 57.1% received it during their primary education (22.9% during both). Other sources of education are outlined in Table 4.

**Table 4:** Sources of anti-doping information (in %)^a.^

	Athletes^b^	Coaches
NADO	67.5	82.9
WADA	46.0	51.4
National sport federation	35.7	62.9
IPC	27.0	31.4
National Paralympic Association	25.4	5.7
International sport federation	8.7	22.9

^a^ It was possible to give multiple answers.

^b^ Three athletes (2.4%) said to receive information from their team doctor.

Of those who received education, the majority perceived it as trustworthy (athletes: 61.9%; coaches: 65.7%) and worthwhile (coaches: 60.0%). Furthermore, and rather in general, sport authorities' legitimacy to be entrusted with anti-doping issues was rated moderately, M = 4.32 ± 1.36 by athletes and M = 3.91 ± 1.19 by coaches (on a 7-point Likert scale). No significant differences in sex were found for athletes (*p *= 0.90, *d *= 0.03). Several comments were added in the open-ended section of the survey. For example, a very prominent issue seems to be the area of doping tests with a call for “*international fairness”* as athletes abroad experience a “*maximum of one doping test per year with forewarning”*. There is a “*lack of testing post competition”* as one athlete won two gold medals at the Paralympic Games and was “*only tested* via *urine sample”* once. Therefore, “*more tests need to be done”*, but anti-doping personnel (excluding the doctor) are seen as “*overloaded/insufficient”* at international competitions. One coach underpins that there are “*far too few doping controls”* and refers to the IPC statistics. Another coach became aware during the survey that his “*knowledge about anti-doping regulations in other nations (how much testing, etc.) is very limited”*.

In sum, athletes agreed that they were well informed of topics identified in the WADC 2021 and ISE 2021 (M = 5.52 ± 1.21, on 7-point Likert scale). No significant differences were found in knowledge across the topics for athletes (*p *= 0.84, *d *= 0.04).

Coaches' perceived knowledge about their rights and responsibility according to the WADC was moderate, M = 5.09 ± 1.25 (on a 7-point Likert scale).

### 3.4. Coaches: engagement in doping prevention, roles, and responsibilities within the WADC, and doping confrontation efficacy

In general, coaches positively answered the statements with respect to their engagement in doping prevention. There were no significant differences between the countries except for talking about doping substances and methods. With respect to this item, UK coaches indicated lower agreement compared to the rest (for details, refer to Table 5).

**Table 5:** Coach engagement in doping prevention in %.

	All	Germany	UK	Austria	Switzerland
	(*n* = 35)	(*n* = 19)	(*n* = 4)	(*n* = 3)	(*n* = 9)
Discuss physical and psychological problems with your athletes	100	100	100	100	100
Discuss winning or losing with your athletes	71.4	73.7	50	100	66.7
Talk about doping substances and methods with your athletes	77.1	78.9	25 ^a^	100	88.9
Is doping prevention a relevant topic in your training routine	62.9	78.9	50	66.7	33.3
Prepare athletes for doping controls	77.1	73.3	100	100	66.7
Encourage your athletes to take nutritional supplements	28.6	36.8	50	0	11.1

^a^ Significant difference between UK and the rest (*p* = 0.50).

Figures represent “yes”.

Overall, coaches showed a high agreement towards their pre-defined roles and responsibilities as based on the WADC, a mean >6 (on a 7-point Likert scale) in all items, with no differences between the countries (0.62 < *p *< 0.91; 0.02 < *eta^2^^ ^*< 0.06, for details, refer to Table 6).

**Table 6:** Coaches’ agreement of their roles and responsibility according the WADC 2015 (1 = strongly disagree; 7 = strongly agree).

	M ± SD
I am knowledgeable of and comply with all anti-doping policies and rules adopted pursuant to the Code and which are applicable to them or the athletes whom they support.	6.38 ± 0.94
I cooperate with athlete testing programs.	6.31 ± 1.51
I use my influence on athlete's values and behaviour to foster anti-doping attitudes.	6.56 ± 1.13
I disclose to my national anti-doping organisation and international federation any decisions by a non-signatory finding that I have committed an anti-doping rule violation with the previous ten years.	6.69 ± 0.85
I cooperate with anti-doping organisations investigating anti-doping rule violations.	6.81 ± 0.74
I do not use or possess any prohibited substance or prohibited method without valid justification.	6.61 ± 1.38

In relation to coaches’ confidence in their ability to confront doping, all ratings can be considered high and in the following order (highest rating to lowest rating): “Legitimacy”: M = 6.19 ± 0.96; “Initiation”: M = 5.78 ± 1.17; “Outcomes”: M = 5.56 ± 1.17; “Resources”: M = 5.55 ± 1.10; “Intimacy”: M = 5.32 ± 1.13 (all on a 7-point Likert scale). There were no significant differences between the countries.

## 4. Discussion

The current research aimed to describe a sample of para-athletes and parasport coaches, to understand their perceptions of ADRVs and anti-doping responsibilities. Additionally, informed by previous research in Paralympic and Olympic sport, the concept of norms, anti-doping education, knowledge, and perceived legitimacy as well as coach engagement, their roles and responsibilities, and doping confrontation efficacy were investigated. Overall, there were no marked differences between para-athletes and parasport coaches compared to Olympic athletes and coaches and only little differences between the countries. Most of the athletes and coaches were educated, all showed a low doping willingness, knew about their roles and responsibilities, and considered doping more of a problem in other sports and not in their own. Interestingly, classification cheating was considered a form of doping and seems to be an important issue for athletes and coaches, especially within the UK sample. Comparable with studies from research about Youth Olympic athletes (47), but a rather dissatisfying finding in relation to the ISE (2021), was the fact that for 33.3% of the athletes a doping control was their first experience with anti-doping. Coaches' engagement with doping prevention activities and their perceived efficacy to confront doping-related matters appear to be higher compared to Olympic coach samples included in previous research.

### 4.1. Descriptive norms—doping is a relevant issue in Paralympic sport

Independent of their role, there was no difference in how athletes and coaches perceived doping in Paralympic sport. In detail, endurance and strength sports are considered by both coaches and athletes to be particularly affected by doping compared to skilled sports. This perception of high-risk and low-risk sport disciplines was previously shown in Paralympic (5, 6) and able-bodied sport (3, 48, 49) and aligns with the finding in able-bodied sport that coaches perceive doping as less prevalent in low-risk sports (= skilled sport) than high-risk sports (= endurance and strength sport) (50). Interestingly, the findings of this study also show that “the others” are perceived to be more involved in doping than oneself. This perception is typical for ethically questionable behaviour and often reported by able-bodied sport coaches (30, 50, 51), and recently by para-athletes (6) too. This gap might be explained by the false consensus effect in doping (52). Notably, for the first time, our results indicate that female para-athletes estimate the prevalence of doping significantly higher compared to male athletes in almost all statements.

The overall perception of about 20% doping prevalence is in strong contrast to WADA's findings of about 1% (8–14). The same phenomenon can be found in able-bodied sport. A review study of doping in able-bodied elite sport concluded that 14%–39% of athletes have probably been involved in doping (15, 16). An explanation of the difference might be the underestimation of the testing results, that might be rooted in a lack of overall numbers of tests. In line, 16.7% of athletes in this survey indicated they had never been asked for a doping control although all of them have been members of a registered testing pool. This is supported by previous qualitative research in which more, and with a fairer international balance, doping tests have been requested (6, 7). There is no clear answer to this, on the one hand it could be that indeed too few tests are done and we should increase the testing. One reason why more doping tests might not have been implemented evenly so far might be the financial resources of the responsible national anti-doping organisations, as doping tests are very expensive to conduct (6). On the other hand, it could also be that the current self-reported numbers are over-exaggerated and the true value is closer to the testing figures than we believe. In support of this, a recent evidence synthesis on doping prevalence in sport concludes that the current knowledge on prevalence relies upon “weak evidence” and thus, these numbers, especially the self-reported numbers, should be treated with care (53). Either way, communicating prevalence numbers to athletes has consequences, which we should be aware of as the false consensus effect might lead to a wrong justification of doping if athletes believe that everyone else is doing it anyway. In this regard, the focus might be given to female athletes, as they seem to estimate the prevalence numbers higher than male athletes which in turn could have an effect on their doping behaviour. Communicating changes in testing figures, at least, would respond to athletes' articulated gap of the need for increased and fairer testing and they are certainly not overestimated.

### 4.2. Anti-doping education with room for improvement

The ISE considers values-based anti-doping education significant in athlete's careers to prevent doping (35). Education programmes should foster a strong anti-doping stance of athletes, coaches, and other support personnel to create an overall culture of clean sport (54). Around two-thirds of athletes and coaches indicated that anti-doping education was their first contact with the topic. This aligns nicely with the main objective of WADA's ISE (35), which states that athletes' first contact with anti-doping should be via education. Anti-doping education is supposed to introduce anti-doping controls, explain athletes' rights during a control and the exact procedure to be prepared—all relevant information to be received prior to the first doping control. Yet almost 30% of athletes who indicated that the testing procedure was their first contact with anti-doping can still be considered surprisingly high because all participating athletes stem from four countries of the Global North in which the implementation of anti-doping education is thought to be of a high standard (36). Thus, there is the need to continue to work on the reach of education to ensure that the prevalence of athletes whose first contact with anti-doping is through education can be increased beyond the two-thirds reported. Aligned with that, we suggest starting anti-doping education as early as possible in athletes' careers. Early in that sense does not necessarily mean “young” age, especially in disabled sport, also shown in our sample, the average age of the athletes is higher as some entered the professional disabled sport system after an accident later in their life, for example. As such, we would recommend continuing to work on ensuring that every athlete, once entering the professional (para) sport system receives anti-doping education to make sure this encounter is prior to his/her first doping control.

Independent of whether education was their first encounter with anti-doping or not, a positive finding of this research is the fact that many of the coaches and athletes received 1–3 anti-doping education sessions throughout the last year and reported to have perceived the content as trustworthy and worthwhile. Unfortunately, 28.6% of athletes did not attend any anti-doping education sessions within the last year. Interestingly, 33% of these were those athletes who indicated that doping controls were their first encounter with anti-doping. Overall, 28.6%, to us, appears to be a high number, given the introduction of the ISE in 2021, which mandates anti-doping education for all athletes, especially when athletes are part of a testing pool. Even though we asked about the last 12 months, that is not excluding any education received before, in high professional sport, we would expect education provided continuously and not only selectively. Yet, similar numbers were reported for UK coaches in able-bodied sport, where almost one in four of the coaches had never learned about anti-doping (55).

In the current research, athletes indicated receiving their anti-doping information from their respective NADO, followed by WADA and their national sport federations. Similar, but not identical, coaches stated that their NADO is the most important source with their national sport federation in second place and WADA in third place. With slight differences, both athletes and coaches rely on their respective NADO, WADA and their national sport federations to receive proper and up-to-date anti-doping education. Consequently, it is very important to supply the named organisations/federations with all required resources (e.g., financial or personnel) to educate all involved stakeholders. It has been shown in previous studies that interviewed Paralympic athletes and parasport coaches perceived anti-doping education worldwide as imbalanced and not on the same standard (6, 7). Within this study, we included Paralympic athletes and parasport coaches of countries of the Global North (UK, Germany, Switzerland, and Austria). Therefore, it would be of high interest if Paralympic athletes and parasport coaches of the Global South agree with the presented findings of the chosen source of information or if their ranking would differ. This question could be a focus in future research to guarantee access to anti-doping knowledge for everyone on the same standard, independent of the country they come from.

### 4.3. Coach engagement in doping prevention, their perceived roles and responsibilities, and efficacy to confront doping behaviour

Coaches' role to support athletes' anti-doping behaviour and implement education sessions is one part of their roles and responsibilities. Within the study, parasport coaches indicated that they do not only feel very well informed about their roles and responsibilities according to the WADC but also revealed that they take those roles and responsibilities very seriously. Furthermore, the coaches showed a high agreement to engage in doping prevention activities. In line, in a sample of Austrian abled-bodied coaches, Blank, Leichtfried (56) found that perceived knowledge about anti-doping is positively associated with engagement in doping prevention. In contrast, independent of their knowledge, a recent study with interviewed parasport coaches concluded that coaches recognize the importance of their role in anti-doping education, but see other stakeholders (e.g., their NADO) responsible for its implementation (7). This aligns with many studies in able-bodied sport (30, 50, 51, 57). Thus, we strongly believe that education needs to raise the matter of responsibility with coaches with respect to what they could do in doping prevention and what others around them might do. This eventually encourages a more intentional and coordinated approach to anti-doping across multi-disciplinary teams (58, 59). At the same time, sports federations who employ coaches should make it clear what they expect of coaches and provide them with appropriate support to facilitate actions that align with these expectations (58–60).

One important resource beyond perceived knowledge that is described in the literature is the doping confrontation efficacy (DCE) of coaches (45). Boardley, Grix (61) could confirm the relevance of all five dimensions with respect to increased confrontation behaviour. Furthermore, athletes’ perception of coaches' doping confrontation efficacy seems to play an important role with respect to athletes' susceptibility to doping intentions and unintentional doping (61) as well as with respect to athletes' anti-doping attitude (60). The latter is especially predicted by the subscale of *intimacy*. In this vein, even though the average confidence of all respective scales was above five (out of seven), the intimacy scale had the lowest mean in this sample of parasport coaches. As reported by Boardley et al., coach education is a key antecedent of DCE. Especially, the dimension of legitimacy is built on the establishment of legitimate grounds for confronting doping behaviour. It can be expected that the perceived knowledge about the topic must be high enough for coaches to engage in this behaviour. As this was, to the best of our knowledge, the first study investigating DCE in parasport coaches, we have no comparison. However, it seems that the DCE in our sample is high and aligned with the high perceived knowledge about anti-doping, a factor associated with DCE. This is encouraging, yet it would be interesting to test the associations of coach engagement and perceived knowledge, mediated by DCE in future studies.

In sum, it appears that parasport coaches take their role in doping prevention more seriously compared to able-bodied coaches and perceive their knowledge about it quite well. In line, the sample of this study displayed high confidence in confronting doping and high agreement towards engaging in doping preventive behaviour. This fact might be important for anti-doping organizations who would not have to convince the coaches about their roles anymore but can directly build on these resources by focusing on keeping up the education level and thus, the perceived knowledge about anti-doping, fostering the intrinsic motivation to consider anti-doping a coach responsibility and providing them the room and the time to implement anti-doping related content into their everyday practice.

### 4.4. Classification cheating as a broader integrity issue in Paralympic sport

An interesting finding, that confirmed findings from the interview studies performed prior to this study, was related to classification cheating. A majority of both Paralympic athletes and parasport coaches considered “classification cheating for a competitive advantage” as a form of doping. So-called “classification cheating” has been reported in previous research (6, 7, 62), although it is not an ADRV according to the IPC anti-doping Code. Therefore, cheating on classification is not a sanctioned doping behaviour and cannot be defined as such. Yet, it is important for athletes and coaches, who considered it a threat to parasport integrity. The current problem is that athletes might intentionally misrepresent their abilities (e.g., exaggerate their disability or not prove their ability at their best), and therefore positively influence the class in which they will compete. This behaviour of “classification cheating” puts some athletes in a better position and disadvantages those who are correctly classified. This behaviour might lead to increased pressure for co-competitors to engage in doping to level the playing field again (6). In addition, manipulation of one's classification undermines the set values by the IPC, namely, equality, courage, inspiration, and determination (55).

Although there is no research that explicitly explores classification cheating, other studies have analysed the currently applied classification methods (63), potential improvement for classification (64–66), or the question of whether the classification system is fair enough to create an even competition (67, 68). Summarizing, the made efforts move the classification system in the right direction to accurately classify athletes and following, enable fair competition. On top of that, more efforts are being made to continuously improve/adapt the given rules. In 2018, the IPC Governing Board released guidance on how to classify athletes with visual impairment (69). Other sport federations have followed suit and revised their current classification rules. For example, in 2019, the International Tennis Federation introduced a new classification system within Wheelchair Tennis, in which they identified a need to be classified by a professional and not by the athletes themselves. One way to protect fair classes and the integrity of Paralympic sport could be to think about sanctions for classification cheating similar to sanctions for an ADRV, as proposed previously (6, 70). However, given the current rules, future general education initiatives in sport should, instead of including it in anti-doping education, emphasize that classification cheating as well as, for example, technical cheating is fraud and leads to unfair competitive advantages but is, currently, not considered doping. At the IPC, the position of the World Para Sports Classification Project Senior Manager is responsible for overseeing the classification process, revising current classification systems, and educating on classification. There even is a specific role of a classification education manager. Therefore, there is no need for anti-doping organisations to take on this responsibility as part of their programmes; though, perhaps they could coordinate their efforts, and share their learnings, with individuals responsible for classification (given that classification is being perceived as a “doping” problem by athletes and coaches).

### 4.4. Limitations

The most common and acknowledged limitation within the issue of (anti-)doping is social desirability. Therefore, we cannot be certain that all participants answered according to what they perceive/believe/think rather than what might be socially accepted. To reduce this potential behaviour, we assured several times that the survey was anonymous, that we could not trace answers, and that participants could stop answering at any time and quit without consequences. Another limitation might be the long period of time in which we collected data. The planned time frame of three months was made impossible due to the postponed Paralympic Games. Due to this major event for both Paralympic athletes and parasport coaches, our target group were often busy with their preparations for the Games and did not have time to complete the survey. Therefore, we prolonged the survey period and asked coaches and athletes several times. However, we tried our best to motivate as many Paralympic athletes and parasport coaches as possible and perceive our final sample as sufficient since it makes a unique contribution to Paralympic research so far. The third limitation is related to two selection biases that are a result of our convenience sampling approach. All respondents stem from four countries of the Global North and thus, our results cannot be generalized. Therefore, more countries around the world should be included in further research to test the repeatability and generalisability of our findings. Additionally, it can be expected that athletes with a higher willingness to dope would not participate in such a study and are thus, excluded from the data.

### 4.5. Conclusion and practical implications

As expected, the results of this study support the hypothesis that there are similarities in doping (e.g., prevalence, sport disciplines) and anti-doping (education, knowledge, perception) between Paralympic and Olympic sport. Thus, sport organisations/NADOs in Paralympic sport could use synergies and work closer together with those organisations in Olympic sport. This means that similar approaches to anti-doping education could be adopted. Endurance and strength sports were viewed as higher risk and could therefore potentially receive targeted testing and education. Future research could especially draw attention to those sport disciplines regarding the nature and effectiveness of their education programmes and testing strategies. Associated with that, future research should include participants from the Global South to assess whether findings would be similar compared to this presented sample.

Key implications of our research relate to ensuring a balanced communication of doping prevalence numbers and testing figures among para-athletes (as with able-bodied athletes). Furthermore, we encourage all international sport organizations to continuously keep the effort high to ensure athletes are educated about anti-doping before they are tested. This might mean implementing anti-doping education early in athletes' careers, with early representing at a young age, or with early representing when they enter the field of professional (para) sport. Aside from the athletes, coaches perceived knowledge is high and they feel educated well. As this seems to be associated with coach engagement in anti-doping and increased levels of DCE, we furthermore encourage responsible organizations to also ensure there is enough space and time for the coaches to apply their knowledge. It seems that in parasport, different compared to able-bodied coaches, anti-doping organizations do not have to convince the coaches about their roles (i.e., being responsible for anti-doping education) anymore but can directly build on these resources by focussing on keeping up the education level and thus, the perceived knowledge about anti-doping, fostering the intrinsic motivation to consider anti-doping a coach responsibility and providing them the room and the time to implement anti-doping related content to training routines. With respect to classification cheating, we would emphasize that classification cheating is fraud and leads to unfair competitive advantages, but is, currently, not considered doping. Thus, it should be part of more general sports integrity education and not necessarily part of anti-doping education. Overall, it seems that there are few differences between parasport and able-bodied sports and thus, instead of putting a lot of money into the development of specific parasport anti-doping education, responsible organisations could use the existing programmes in Olympic sport and only adapt special content (e.g., boosting) which is unique to Paralympic athletes. This could save a lot of money which could be used to draw more attention to the sport integrity of Paralympic sport.

## Data Availability

The raw data supporting the conclusions of this article will be made available by the authors, without undue reservation.
